# Food and alcohol disturbance among young adults during the COVID-19 lockdown in Italy: risk and protective factors

**DOI:** 10.1007/s40519-021-01220-6

**Published:** 2021-05-29

**Authors:** Sara Pompili, Daniele Di Tata, Dora Bianchi, Antonia Lonigro, Marta Zammuto, Roberto Baiocco, Emiddia Longobardi, Fiorenzo Laghi

**Affiliations:** 1 Department of Social and Developmental Psychology, Sapienza University of Rome, via dei Marsi, 78 00185 Rome, Italy; 2 Department of Human Sciences, European University of Rome, Rome, Italy; 3 Department of Dynamic and Clinical Psychology, and Health Studies, Sapienza University of Rome, Rome, Italy

**Keywords:** Drunkorexia, Food and alcohol disturbance, COVID-19, Emotion regulation, Social support, Living arrangement

## Abstract

**Purpose:**

The COVID-19 lockdown measures have had a significant impact on risk behaviors as alcohol use and disordered eating. However, little is known about a serious health-risk-behavior named “food and alcohol disturbance” (FAD), characterized by engaging in dysfunctional eating on days of planned alcohol consumption. The aim of the present study was to investigate potential factors that may have put young adults at risk or protected against FAD during the COVID-19 lockdown.

**Methods:**

A sample of 447 young adults (280 females, 167 males; range 18–26) completed an online survey during the country’s nationwide lockdown composed of self-reported measures assessing FAD behaviors, alcohol consumption, compensatory behaviors, eating and weight concerns, social support, emotion regulation strategies, and living arrangement.

**Results:**

Our findings showed that FAD was significantly and positively correlated to alcohol consumption, use of laxatives, self-induced vomiting, eating and weight concerns, and expressive suppression, and negatively correlated to social support and living with family. Hierarchical regression analysis revealed that alcohol consumption, eating concern, and expression suppression positively predicted FAD, while social support and living with family were negative predictors.

**Conclusions:**

Our results suggest that during the COVID-19 lockdown, preoccupation with eating and the use of expressive suppression may have increased vulnerability to FAD; conversely, perceived social support and living with family may have been a source of protection against this dysfunctional behavior.

**Level of evidence:**

Level V, descriptive study.

## Introduction

The coronavirus disease (COVID-19) that first appeared in China, has rapidly spread worldwide becoming a global pandemic [1]. To mitigate and manage the health emergency, containment measures have been introduced by governments of countries affected by COVID-19. Italy was the first European country to be deeply hit by the pandemic and to adopt a nationwide lockdown that was ordered on March 9th, 2020 in an attempt to control the viral transmission [2]. Lockdown measures involved that people stay home except for emergencies, shopping for basic necessities, health problems, and to go to work (in the case smart-working practices were not possible). Schools were temporary closed, as well as nonessentials shops, and any form of gathering of people in public places or locations accessible to the public was prohibited. Although these restrictions were necessary to contain the transmission of the virus and the pressure on the healthcare system, available evidence has highlighted the significant impact that lockdown measures have had on people’s psychological well-being and health-related behaviors [3–6]. Indeed, it was found that the prolonged and mandatory home confinement has had an effect on the engagement of dysfunctional behaviors, such as alcohol use [7, 8] and disordered eating [9–11]. Specifically, it has been noted that during the COVID-19 lockdown, individuals have used alcohol and food in an attempt to respond to the negative affect and stress experienced [8, 10]. Indeed, long isolation and social distancing may increase negative emotions and stress, and disordered eating and alcohol consumption may serve as a way of coping with distress related with an uncertain situation as the pandemic [8, 12].

However, to our knowledge, no study so far has investigated an emerging and serious combination of disordered eating and alcohol consumption named “food and alcohol disturbance” (FAD) [13] during the lockdown, and the potential factors that may have been involved in protecting or increasing vulnerability to this behavior. Sometimes referred to also as “drunkorexia” [14, 15], FAD concerns a variety of unhealthy eating behaviors in which people engage in preparation or in response to alcohol consumption [16], and can include calorie restriction, fasting, self-induced vomiting, use of laxatives and/or diuretics, and excessive exercising [17–20]. Indeed, FAD is typical of individuals who choose to engage in diet-related behaviors before or after drinking large amounts of alcohol, to compensate for calories consumed through alcoholic beverages for the fear of gaining weight, or to enhance the intoxicating effects of alcohol [18, 21, 22]. Recently, researchers have proven that FAD is predicted by poor body esteem, high sensation seeking [23, 24], and drive for thinness [25], thus suggesting that people may use this at-risk behavior to pursue an idealized body image. Moreover, in recent studies [26], FAD appeared to be driven by motivations analogous to eating and substance use disorders: applying the Cooper’s alcohol motivation model, researchers found that enhancement and conformity motives were both related with compensatory behaviors, suggesting that young adults may limit their caloric intake on drinking days to comply with social expectations and to be accepted by others [26].

FAD behaviors are a quite frequent among young people. Before COVID-19 outbreak, from the international prevalence of young adults reported limiting their caloric intake on days they had planned to drink alcohol ranged from 37 to 46% [22, 27, 28]. In Italy, the pre-pandemic occurrence of FAD behaviors was of about 32% in young adults [29] and 13% in adolescents [30].

Gender differences in FAD behaviors are generally inconsistent, as emerged by previous research. Most studies suggest the absence of gender differences in FAD frequency [16, 31, 32]. However, some authors found that women (vs. men) are significantly more likely to engage in restricting behaviors before drinking alcohol [33, 34], and are also more likely to engage in FAD behaviors to enhance alcohol’s effects [20]. Further evidence suggested that men are more likely to work out to manage their caloric intake in preparation or in response to alcohol consumption, whereas women usually prefer alternative strategies, such as skipping meals and purging [35]. The repeated combination of alcohol consumption and food restriction represents an important health issue because of its association with several detrimental consequences, such as memory lapses, psychological distress, and tendency to engage in at-risk behaviors [27, 36, 37].

Existing literature has repeatedly underlined that alcohol and food are substances that individuals may use in an attempt to deal with and manage negative and stressful emotional experiences they are unable to regulate with more adaptive strategies [20, 38]. Indeed, it has been noted [39–41] that both disordered eating and alcohol use are related to difficulties regulating emotions and managing situations involving negative affect. Specifically, it has been showed that the lockdown measures may exacerbate such difficulties in emotion regulation, which may increase the vulnerability for eating disorder symptoms [42]. In addition, a significant and recurring use of maladaptive emotion regulation strategies, as expressive suppression, has been found to be a risk factor for both alcohol use and dysfunctional eating [39, 43, 44]. Eating and weight concerns are also factors associated with a greater risk for dysfunctional eating behaviors [45]. Indeed, preoccupation with food, calories, and the fear of gaining weight may predispose people to dieting and purging behaviors to gain a sense of control on external events or internal feelings [46, 47]. An increase in preoccupation with eating and weight has been showed during the pandemic and dysfunctional eating behaviors may function as a means of regain control in the current crisis characterized by uncertainty and inconstancy [48].

Moreover, previous studies have indicated that even living arrangement seems to influence dysfunctional eating and drinking behaviors [18, 49, 50]. Specifically, living alone during the COVID-19 pandemic in a context of loneliness and isolation may increase vulnerability for health-risk behaviors [12, 51], affecting the occurrence of both unhealthy eating behaviors and alcohol use [49, 50, 52]. Conversely, living with family has appeared to be a protective factor that may help control the engagement in these risk behaviors [18, 53]. In the same way, the perception of being understood and supported in stressful situations has also been found to be a protective factor against dysfunctional eating and alcohol consumption [54, 55]. For instance, it has been highlighted [54] that tangible social support, as expecting help in case of getting sick or emergency, and the possibility of being able to rely on someone to talk to or relax with, may protect against alcohol involvement. Social support has also been found to play a role in mitigating the impact of different stressors in the context of epidemic diseases [56].

The aim of the present study was to investigate potential risk and protective factors that during the COVID-19 lockdown may have been involved in the engagement of FAD among young adults. Specifically, the current research was aimed at investigating the association of FAD with alcohol consumption, compensatory behaviors, emotional regulation strategies, eating and weight concerns, social support perceived, and living arrangement during the lockdown period; furthermore, the research was aimed at exploring the role of preoccupations with eating and weight, social support, living alone and living with family, and emotion regulation strategies in predicting FAD. To our knowledge, this was the first study to be focused on FAD and to explore factors that may have increased the vulnerability or, conversely, have protected young people against this health-risk behavior during the lockdown. In line with prior research [47, 48], we hypothesized that eating and weight concerns may be thoughts that could drive young adults to engage in FAD behaviors in an attempt to gain control over their internal states and external circumstances. Furthermore, consistent with previous studies [50, 52], we hypothesized that living alone during the pandemic lockdown, in a condition of prolonged social isolation, may be a risk factor for engaging in FAD, as well as the use of dysfunctional strategies to regulate emotions, as existing literature suggests [8]. Conversely, living with family and perceiving social support may function as protective factors against FAD, in line with available evidences [53–55].

## Methods

### Participants and procedure

The sample consisted of 447 young adults (280 females and 167 males) with a mean age of 23.00 years (SD = 1.93; range 18–26). They were required to live in Italy during the COVID-19 lockdown to participate in the study. In terms of their job status, 62% were students, 20% were employees, 7% were unemployed, 6% were trainees, and 5% were self-employed. Regarding participants’ job condition during the lockdown, 46% reported that their work was fully or partially suspended due to the lockdown measures, 32% reported that they did not have a job, and finally, 22% continued to work without any suspension. A snowball sampling method was used for the recruitment of the participants for the survey. Data were collected in April and May, 2020 during the country’s nationwide lockdown, using an anonymous online survey, which was shared through the University website. Prior to participation in the study, individuals were invited to read the informed consent form and explicitly give their agreement to take part to the research. Of the individuals who agreed to participate in the study, 96% fully completed all the required measures. The anonymity and confidentiality of the results, as well as the voluntary nature of the research participation were guaranteed. The participants were informed that they could leave the study at any time. They did not receive any reward to take part in the study. This survey was reviewed and approved by the Ethics Commission of the Department of Developmental and Social Psychology of Sapienza, University of Rome.

### Measures

#### FAD

To assess FAD during the COVID-19 lockdown, participants were asked to answer a single item used in previous studies [22, 30, 57], indicating during the lockdown period, how often they restricted their food, calories, or carbohydrates on days they planned to drink alcohol to avoid weight gain. Response options ranged from 1 (never) to 5 (often). According to Laghi, Bianchi et al. [57] and Pompili and Laghi [30], this measure provided a valid indicator of FAD behaviors.

Following the procedure suggested in prior research [30], young adults who engaged in restrictive eating or drinking days were categorized as “restrictors”, whereas those who reported never engaging in FAD behaviors were categorized as “non-restrictors”. We considered as indicating no FAD tendencies, only non-reported restrictive eating in response to alcohol use (answer option 1 = never).

#### Alcohol consumption

Alcohol Use Disorders Identification Test-Consumption (AUDIT-C) is brief alcohol screen used to identify a risky or harmful alcohol consumption. It is the brief version of the Alcohol Use Disorders Identification Test (AUDIT) [58], from which it takes only the first three questions, and assesses different dimensions of alcohol consumption: (1) “How often do you have a drink containing alcohol?”; (2) “How many drinks containing alcohol do you have on a typical day when you are drinking?”; (3) “How often do you have six or more drinks on one occasion?”. The score for each response ranges from 0 to 4 points. The minimum final score is 0, and the maximum is 12. AUDIT translation has been validated by Piccinelli et al. [59], and preliminary analyses of the short version of AUDIT have demonstrated its validity and effectiveness as a screening test for problem drinking in the Italian context [60].

#### Compensatory behaviors

The use of inappropriate compensatory behaviors to offset caloric intake during the lockdown period was investigated through the administration of three items, which assessed the frequency of the following behaviors: fasting, self-induced vomiting, and use of laxatives. Compensatory behaviors items were scored from 0 to 6 based on the frequency the participant engaged in the three behaviors. Participants were asked to respond using a 4-point Likert scale (0 = never; 2 = sometimes; 4 = weekly; 6 = daily). These three items were selected for the study because of their ease of administration and briefness, along with their effectiveness in assessing the frequency of compensatory behaviors among young people, as suggested in previous studies [20, 57].

#### Eating and weight concerns

To assess eating and weight concerns during the lockdown period, two items used in previous studies [20] were administered. The items asked participants to indicate their degree of preoccupation with eating (item 1) and weight (item 2) using a 4-point Likert scale (0 = never; 2 = sometimes; 4 = weekly; 6 = daily). Self-reported weight and height were used to calculate Body Mass Index (BMI).

#### Living arrangement

Two items were used to assess the living arrangement, asking participants to indicate both the number and type of persons in the household (“Family”, “Roommates”, “Partners”, “Friends”, “Other”) during the COVID-19 lockdown. For the purpose of the study, in relation to the number of households, participants who reported to live alone during the lockdown were coded with “1”, whereas participants who lived with other people were coded with “0”. In respect of the type of households, participants who indicated to live with their family were coded with “1”, while participants who lived with other types of persons were coded with “0”.

#### Social support

Two designed ad hoc items were used to evaluate the social support perceived during the lockdown: “Think about people you keep in contact with on a daily basis. Do you feel that they can understand and / or support you in case of need?”. This question was referred to both in-person contacts (item 1) and distance contacts (item 2). Participants responded to items on a 3-point Likert scale: 0 (No or I do not have any contact), 1 (Only someone), 2 (Yes, most of them). The two items were summed to yield a total score of perceived social support.

#### Emotion Regulation Questionnaire

The Emotion Regulation Questionnaire (ERQ) [61] is a 10-item instrument designed to assess two Emotion Regulation strategies: Cognitive Reappraisal (6 items) and Expressive Suppression (4 items). Each item is rated on a 7-point Likert-type scale ranging from 1 (strongly disagree) to 7 (strongly agree). The Italian version of ERQ has demonstrated to have good psychometric properties [62]. In the present study, internal consistency coefficients were satisfactory for both Cognitive Reappraisal (*α* = 0.80) and Expressive Suppression (*α* = 0.75).

### Statistical analysis

All data analyses were conducted using SPSS Statistics Version 25.0. Preliminary, consistent with prior research [36, 63], gender differences were investigated by conducting a series of one-way analyses of variance (ANOVAs) on the study variables: FAD, alcohol consumption, compensatory behaviors (fasting, vomiting and use of laxatives), living arrangement (living alone and living with family), social support, eating and weight concerns, and BMI self-reported. We carried out a multivariate analysis of variance (MANOVA) on Emotion Regulation dimensions. For this multivariate analysis, Wilks’ λ criterion was used. Next, Chi-square tests were performed to investigate gender and age differences between restrictors and non-restrictors; for comparative purposes, the sample was divided into two age groups (18–22 years vs. 23–26 years). To examine differences between the two groups on study variables, a series of ANOVAs and a MANOVA were carried out. To protect from Type I error, *p* values for the significance of ANOVAs were adjusted using Bonferroni correction for multiple comparisons (*p*-adjusted value = 0.005). Partial eta-squared values were calculated as a measure of effect size, and results were interpreted using Cohen’s [64] guidelines for determining small (0.01), medium (0.06), and large (0.14) effects.

Furthermore, we performed Pearson’s correlations to examine the relationship among the study variables: FAD, alcohol consumption, compensatory behaviors, living arrangement, social support, eating and weight concerns, Emotional Regulation dimensions, gender, age, and BMI. A hierarchical regression analysis was conducted to assess the contribution of the key variables in predicting FAD. The regression analysis was conducted in five steps with FAD as dependent variable: gender (females = 1, males = 0), age, BMI, and alcohol consumption were entered as covariates in step 1, to control for their potential confounding effect, as prior research highlighted an association between FAD and these variables [22, 65]. In step 2, living arrangement (living alone and living with family) was entered to test its main effect controlling for gender, age, BMI, and alcohol consumption. The perceived social support was entered in step 3 to test its predictive role in FAD, controlling for the independent effects of the covariates and living arrangement. In step 4, eating and weight concerns were added to measure their main effects controlling for the covariates, living arrangement, and social support. Finally, Emotion Regulation dimensions were entered in step 5 to test their predictive power in FAD, controlling for the effects of the covariates, living arrangement, social support, and eating and weight concerns.

## Results

### Preliminary analyses

#### Gender differences

One-way ANOVAs showed a significant difference between males and females on Eating concern, *F* (1, 445) = 12.28, *p* = 0.001, *η*_*p*_^*2*^ = 0.03 and weight concern, *F* (1, 445) = 17.83, *p* < 0.001, *η*_*p*_^*2*^ = 0.04, where females showed a higher mean score than males. A significant difference was also found for BMI, *F* (1, 445) = 9.41, *p* < 0.01, *η*_*p*_^*2*^ = 0.02. Conversely, the two groups did not show any significant difference on FAD, *F* (1, 445) = 2.41, *p* = 0.12, *η*_*p*_^*2*^ = 0.01, alcohol consumption, *F* (1, 445) = 0.65, *p* = 0.42, *η*_*p*_^*2*^ = 0.00, fasting, *F* (1, 445) = 1.45, *p* = 0.23, *η*_*p*_^*2*^ = 0.00, self-induced vomiting, *F* (1, 445) = 1.01, *p* = 0.31, *η*_*p*_^*2*^ = 0.00, use of laxatives, *F* (1, 445) = 1.77, *p* = 0.18, *η*_*p*_^*2*^ = 0.00, living alone, *F* (1, 445) = 2.00, *p* = 0.16, *η*_*p*_^*2*^ = 0.00, living with family, *F* (1, 445) = 0.11, *p* = 0.74, *η*_*p*_^*2*^ = 0.00, and social support, *F* (1, 445) = 0.87, *p* = 0.35, *η*_*p*_^*2*^ = 0.00.

Finally, MANOVA on Emotion Regulation strategies revealed a significant main effect for gender, *λ* = 0.94, *F* (2, 444) = 13.65, *p* < 0.001, *η*_*p*_^*2*^ = 0.06. Results of the univariate tests showed that males and females differed on both Cognitive Reappraisal, *F* (1, 445) = 5.39, *p* < 0.05, *η*_*p*_^*2*^ = 0.01, where females reported a higher mean score than males, and Expressive Suppression,* F* (1, 445) = 19.60, *p* < 0.001, *η*_*p*_^*2*^ = 0.04, where females showed a lower average score than males (Table 1).

**Table 1:** Descriptive statistic (mean, standard deviation, range, frequency, and percentage of study variables) and gender differences

Variable	Total sample (*N* = 447)		Males (*N* = 167)			Females (*N* = 280)			*p* value	*η*_*p*_^*2*^
	M	SD	M	SD	Range	M	SD	Range		
FAD	1.18	0.73	1.11	0.54	1–5	1.22	0.82	1–5	ns	−
Alcohol consumption	1.92	1.75	2.01	1.90	0–10	1.87	1.65	0–8	ns	−
Fasting	0.33	0.92	0.26	0.75	0–4	0.37	1.00	0–6	ns	−
Vomiting	0.06	0.36	0.04	0.27	0–2	0.07	0.41	0–4	ns	−
Use of laxatives	0.07	0.44	0.04	0.34	0–4	0.09	0.48	0–4	ns	−
Social support	3.11	0.97	3.17	0.93	0–4	3.08	1.00	0–4	ns	−
Eating concern	1.85	1.62	1.15	1.50	0–6	2.06	1.66	0–6	0.001^a^	0.03
Weight concern	2.05	1.61	1.64	1.44	0–6	2.29	1.65	0–6	0.000^a^	0.04
Cognitive reappraisal (ERQ)	29.38	5.72	28.57	6.08	6–42	29.87	5.45	12–42	0.042	0.01
Expressive suppression (ERQ)	13.69	4.91	14.99	4.66	4–25	12.91	4.90	4–28	0.000	0.04
BMI self-reported	22.65	3.21	23.25	2.74	17.64–37.44	22.29	3.41	16.56–38.06	0.002^a^	0.02

	*N*	%	*N*	%	*N*	%	*p* value	*η*_*p*_^*2*^
Living alone								
Yes	15	3	3	2	12	4	ns	−
No	432	97	164	98	268	96		
Living with family								
Yes	374	84	141	84	233	83	ns	−
No	73	16	26	16	47	17		

*ns* non-significant

^a^Differences are significant at the 0.005 level (Bonferroni adjusted *p *value)

#### Differences between restrictors and non-restrictors

The sample consisted of 34 (8%) young adults who restricted their food, calories, or carbohydrates on days they planned to drink alcohol to avoid weight gain during the lockdown, and 413 (92%) non-restrictors. The Chi-square tests revealed that restrictors and non-restrictors did not differ in relation to gender, *χ*^2^ (1) = 1.86, *p* = 0.17 and age, *χ*^2^ (2) = 1.87, *p* = 0.17.

Results from ANOVAs showed a significant difference between restrictors and non-restrictors, on alcohol consumption *F* (1, 445) = 12.12, *p* = 0.001, *η*_*p*_^*2*^ = 0.03, self-induced vomiting, *F* (1, 445) = 8.96, *p* < 0.01, *η*_*p*_^*2*^ = 0.02, use of laxatives, *F* (1, 445) = 32.65, *p* < 0.001, *η*_*p*_^*2*^ = 0.07, eating concern, *F* (1, 445) = 21.34, *p* < 0.001, *η*_*p*_^*2*^ = 0.05, and weight concern, *F* (1, 445) = 18.78, *p* < 0.001, *η*_*p*_^*2*^ = 0.04, where restrictors obtained higher mean scores than non-restrictors. Conversely, ANOVAs did not show any difference between restrictors and non-restrictors on fasting, *F* (1, 445) = 6.22, *p* < 0.05, *η*_*p*_^*2*^ = 0.01, social support, *F* (1, 445) = 4.70, *p* < 0.05, *η*_*p*_^*2*^ = 0.01, living alone, *F* (1, 445) = 0.72, *p* = 0.40, *η*_*p*_^*2*^ = 0.00, living with family, *F* (1, 445) = 4.63, *p* < 0.05, *η*_*p*_^*2*^ = 0.01, and BMI, *F* (1, 445) = 0.92, *p* = 0.34, *η*_*p*_^*2*^ = 0.00. Also MANOVA on Emotion Regulation strategies did not find differences between the two groups, *λ* = 0.99, *F* (2, 444) = 2.77, *p* = 0.06, *η*_*p*_^*2*^ = 0.01 (Table 2).

**Table 2:** Differences between non-restrictors and restrictors

Variable	Non-restrictors (*N* = 413)		Restrictors (*N* = 34)		*p* value	*η*_*p*_^*2*^
	M	SD	M	SD		
Alcohol consumption	1.84	1.69	2.91	2.15	0.001^a^	0.03
Fasting	0.30	0.86	0.71	1.38	ns	−
Vomiting	0.04	0.32	0.24	0.65	0.003^a^	0.02
Use of laxatives	0.04	0.31	0.47	1.11	0.000^a^	0.07
Social support	3.14	0.95	2.76	1.23	ns	−
Eating concern	1.75	1.59	3.06	1.58	0.000^a^	0.05
Weight concern	1.96	1.59	3.18	1.49	0.000^a^	0.04
Cognitive reappraisal (ERQ)	29.87	5.45	28.57	6.08	ns	−
Expressive suppression (ERQ)	12.91	4.90	14.99	4.66	ns	−
BMI self-reported	22.61	3.23	23.16	2.89	ns	−

	*N*	*%*	*N*	*%*	*p* value	*η*_*p*_^*2*^
Living alone						
Yes	13	3	2	6	ns	−
No	400	97	32	94		
Living with family						
Yes	350	85	24	71	ns	−
No	63	15	10	29		

*ns* non-significant

^a^Differences are significant at the 0.005 level (Bonferroni adjusted *p *value)

### Correlations among the variables

Pearson correlations were performed to examine the relationship among the key variables used in the present study (Table 3). FAD was significantly and positively associated with alcohol consumption, vomiting, use of laxatives, eating concern, weight concern, and Expression Suppression, and negatively related to living with family and social support.

**Table 3:** Correlations among the variables

Variable	1	2	3	4	5	6	7	8	9	10	11	12	13	14	15
1. FAD	−														
2. Alcohol consumption	0.15**	−													
3. Fasting	0.07	0.06	−												
4. Vomiting	0.10*	0.04	0.29***	−											
5. Use of laxatives	0.28***	0.09*	0.10*	0.31***	−										
6. Living alone	0.04	0.11*	0.12**	0.11*	− 0.03	−									
7. Living with family	− 0.13**	− 0.22***	− 0.09	− 0.06	− 0.07	− 0.42***	−								
8. Social support	− 0.16***	− 0.09*	− 0.10*	− 0.02	− 10*	0.09*	0.48***	−							
9. Eating concern	0.23***	0.01	0.19***	0.08	17***	− 0.04	0.02	− 0.07	−						
10.Weight concern	0.21***	0.04	0.20***	0.15**	0.13**	0.01	− 0.05	− 0.09	0.67***	−					
11. Cognitive reappraisal (ERQ)	0.03	0.01	− 0.01	− 0.03	− 0.05	0.08	− 0.09	− 0.08	0.06	0.06	−				
12. Expressive suppression (ERQ)	0.13**	0.01	0.16***	0.14**	0.10*	− 0.04	0.08	− 0.02	0.08	0.08	0.08	−			
13. Gender (1 = females; 0 = males)	0.07	− 0.04	0.06	0.05	0.06	0.07	− 0.02	− 0.04	0.16***	0.20***	0.11*	− 0.20***	−		
14. Age	0.07	0.04	− 0.06	− 0.06	− 0.06	0.09	− 0.17***	− 0.02	0.01	0.01	0.07	− 0.14**	0.12**	−	
15. BMI self-reported	0.03	0.03	0.01	0.04	0.04	0.06	0.004	0.02	0.17***	0.33***	0.08	0.02	− 0.14**	0.08	−

*p < 0.05

**p ≤ 0.01

***p ≤ 0.001

### Hierarchical regression analysis

Results revealed that in step 1 gender, age, and BMI did not significantly predict FAD; only alcohol consumption emerged as a significant predictor, *β* = 0.15, *p* < 0.001. In step 2, living alone and living with family were added to the model; findings showed that only living with family resulted as a significant and negative predictor of FAD, *β* = − 0.11, *p* < 0.05. Social support entered in step 3 emerged as a negative predictor of FAD as well, *β* = − 0.13, *p* < 0.05. In step 4 eating and weight concerns were entered, accounting for 5% of the variance, with eating concern emerging as a significant predictor of FAD, *β* = 0.17, *p* < 0.01. Finally, introducing Emotion Regulation strategies in step 5, an additional 2% of the variance was explained; results revealed that only Expressive Suppression emerged as a significant predictor of FAD, *β* = 0.13, *p* < 0.01; Cognitive Reappraisal was not found to be related to FAD. Overall, the final model accounted for 12% of the variance (Table 4).

**Table 4:** Hierarchical regression analysis for variables predicting FAD

Predictor	*B*	SE B	*β*	*R*^2^	∆*R*^2^	*df*	*∆F*
Step 1				0.03	0.03	4.442	3.64**
Gender (1 = females; 0 = males)	0.12	0.07	0.01				
Age	0.02	0.02	0.05				
BMI self-reported	0.01	0.01	0.03				
Alcohol consumption	0.06	0.02	0.15**				
Step 2				0.04	0.01	2.440	2.32
Living alone	− 0.13	0.21	− 0.03				
Living with family	− 0.22	0.10	− 0.11*				
Step 3				0.05	0.01	1.439	5.11*
Social support	− 0.10	0.04	− 0.13*				
Step 4				0.10	0.05	2.437	11.96***
Eating concern	0.07	0.03	0.17**				
Weight concern	0.04	0.03	0.09				
Step 5				0.12	0.02	2.435	4.45*
Cognitive reappraisal (ERQ)	− 0.005	0.01	0.04				
Expressive suppression (ERQ)	0.02	0.01	0.13**				

*p < 0.05

**p < 0.01

## Discussion

The aim of the current study was to explore potential factors that could have been a source of vulnerability or protection for engaging in FAD among a sample of young adults during the COVID-19 lockdown. Our results highlighted that 8% of young adults engaged in FAD during the lockdown. Compared to the pre-pandemic-estimates, which showed an occurrence of this behavior of about 32% [29], our data seem to suggest a substantial decrease in the frequency of FAD during the lockdown period. Such difference may be due to the restrictive measures imposed by the COVID-19 lockdown, which did not allow young adults to plan to go out drinking alcohol with their peers. Indeed, FAD represents a socio-cultural practice among young people, and may be influenced by the pressure to conform to peers’ group norms by drinking alcohol, and by the desire to achieve a socially acceptance body shape and weight [34]. Thus, it has been suggested that young people may engage in FAD during social activities, where they are more exposed to peer pressure [30].

As expected, both alcohol consumption and compensatory behaviors were related to FAD during the lockdown, although our results highlighted that only self-induced vomiting and laxatives were associated with FAD. Such finding may be related to the evidence that during the lockdown, individuals have reported difficulties in controlling their eating, and it has been suggested that the continuous access to food, feelings of boredom, and loneliness may have been involved in the inability to manage their diet [10]. Thus, among disordered eating behaviors, young adults may have chosen to use laxatives or vomiting, as they have found difficulties limiting caloric intake, for instance, using fasting to avoid an increase in weight. Furthermore, in line with prior research [20, 45, 48], FAD was associated with eating and weight concerns, although only preoccupation with eating emerged as a significant predictor. Our results seem to indicate that during the lockdown, thoughts about food and calories may have been a risk factor for engaging in FAD. This result supports the notion that young people who engage in FAD impose strict rules on themselves on drinking days, which may include the control of the amount as well as the type of food to consume [16]. For instance, there is recent evidence that young adults tend to eat healthier foods the day after drinking, to cleanse or detoxify their bodies [66]. However, as mentioned earlier, the monitoring and control overeating could have been particularly difficult in this period characterized by staying at home with enhanced availability of food [10], giving rise to great concern. Indeed, it has been noted that preoccupation with eating has increased during the pandemic [48], and FAD may have been used as a way to obtain a sense of control both on the self and on external situations as the pandemic, and the uncertainty it involves. In addition, our findings highlighted that during the lockdown, the use of non-adaptive strategies to regulate emotions, such as expressive suppression, may represent a significant predictor of FAD. Thus, the inability to effectively manage and express emotions may increase vulnerability to FAD. Indeed, previous studies underlined that the use of ineffective emotion regulation strategies, which tends to prolong and intensify negative affect, represents a risk factor for both alcohol use and disordered eating [39, 43]. Specifically, it has been showed that during the lockdown, alcohol and food have been used as a means to comfort and relief form negative emotions [8, 10]. A recent research highlighted that also FAD may be used as a way to cope with aversive affective states young people are not able to manage and express in a more adaptive way; thus, this risk behavior may help reducing or distracting from negative affect experienced during the pandemic lockdown [36]. Conversely, having lived with family during the stay-at-home period seems to be a protective factor against FAD. Indeed, in line with available evidence [18, 53], the presence of family members may be a source of protection which may help young people to control their engagement in disordered eating behaviors on drinking days. Furthermore, it is plausible to assume that living with family during the lockdown may have provided a source of distraction, helping individuals not to use FAD as a way to distract from and cope with negative emotions. In addition, social support resulted as another protective factor. This finding supports the evidence of the beneficial role of social support during a pandemic [56], and in line with previous studies [54, 55], highlighting that the perception of being able to count on a tangible support in case of necessity, and the availability of someone to talk to or relax with, may protect against alcohol consumption and disordered eating.

## Limitations

To our knowledge, this was the first study aimed to investigate potential risk and protective factors that during the COVID-19 lockdown may have been involved in the engagement of FAD among young adults. However, it is important to acknowledge some of the limitations. First, due to the cross-sectional design tested in our study, we were not able to establish the direction of the associations we analyzed; thus, future research could consider the implementation of a longitudinal design to better understand the temporal nature of the variables and to test the potential long-term effects of the risk and protective factors on FAD. Second, our study relied on self-report measures and respondents may have misreported some of the questions of the survey. Third, the study involved a sample of Italian male and female young adults, and thus, our findings might not generalize to non-Italian young adults. Furthermore, we used a single item to assess FAD in line with previous studies [22, 30, 57]. As this item assessed the engagement in FAD for compensating for calories to avoid an increase in weight (which is one of the primary motivations underlying FAD), this measure might have not captured FAD behaviors driven by other motivations, such as enhancing alcohol effects. Thus, this might have affected the number of restrictors in the present study, as well as the use of a snowball sampling, which might have been a potential source of bias. In addition, FAD behaviors might have been impacted by the timing participants completed the survey during the COVD-19 lockdown, for instance, at the very beginning or around the last period. Finally, another limitation of this study was not collecting information about participants’ experiences of symptoms of anxiety and depression during the lockdown and the presence of prior eating psychopathology.

## What is already known on this subject?

Existing evidence has highlighted the great impact that the COVID-19 lockdown have had on the engagement in health-risk behaviors, such as alcohol use and disordered eating. However, to our knowledge, no study has investigated FAD during the lockdown period, and the potential factors that may have protected against or increased vulnerability to this dysfunctional behavior among young adults.

## What does this study add?

The present study revealed that the preoccupation with eating and the use of non-adaptive strategies to regulate emotions, as expressive suppression, may have increased the vulnerability for engaging in FAD during the prolonged stay-at-home period. Conversely, living with family and the perception of social support have resulted to protect against engaging in disordered eating behaviors when alcohol consumption was planned. The findings of our study may have implications both in terms of early identification of young adults at risk for FAD, as well as in terms of prevention, contributing to the implementation of effective programs, which may be focused on helping young adults to develop more adaptive emotion regulation strategies, as being able to tolerate and express negative emotional states, and to strengthen their social support networks.
